# Network Topology of Wing Veins in Hawaiian Flies Mitigates Allometric Dilemma

**DOI:** 10.3390/biomimetics9080451

**Published:** 2024-07-24

**Authors:** Kazuki Sugiyama, Yoshihiro Kubota, Osamu Mochizuki

**Affiliations:** 1 Graduate School of Science and Engineering, Toyo University, Kujirai 2100, Kawagoe 350-8585, Japan; 2 Faculty of Science and Engineering, Toyo University, Kujirai 2100, Kawagoe 350-8585, Japan

**Keywords:** fluid mechanics, insect wing veins, network topology, pipe network, hemodynamics, Hawaiian *Drosophila*

## Abstract

Specific Hawaiian fruit flies have an extra crossvein (ECV) in the wing vein network which connects contiguously with another crossvein and forms a unique cruciform topology. These flies are distinguished by their large wings and their allometrically small vein diameters compared to those of typical fruit flies. Small vein diameters may increase frictional energy loss during internal blood transport, although they lead to an improvement in the wing’s moment of inertia. Our hypothesis was that the ECV’s presence would reduce the hydraulic resistance of the entire vein network. To investigate the hemodynamic effects of its presence, the flow rate of blood and frictional pressure loss within the vein networks was simulated by modeling them as hydraulic circuits. The results showed a 3.1% reduction in pressure loss owing to the network topology created by the presence of the ECV. This vein and its contiguous crossvein diverted part of the blood from the wing veins topologically parallel to them, reducing the pressure loss in these bypassed veins. The contiguity of the ECV to the other crossvein provided the shortest blood transfer route and lowest pressure drop between these crossveins. The results suggest that the presence of the ECV may counterbalance the heightened resistance caused by constricted veins.

## 1. Introduction

Networks of insect wing veins transport insect blood called hemolymph, which is crucial for the nourishment and functioning of the wings. Hemolymph in winged insects typically enters by the anterior veins located at the base of the wing, then circulates through the network of veins, and finally departs through the posterior veins at the base [1,2,3]. The hemolymph is suctioned by pumping organs in the thoracic region [4,5,6]. Some of these organs are referred to as wing hearts, particularly those of most holometabolan insects, including flies [2,4]. The hemolymph in the veins supplies the wing tissue with substances [6] and the wing cuticle with water to maintain its mechanical properties [7,8]. Therefore, the circulatory system can be considered as a fluid-transporting network consisting of conduits and pumps. Previous studies have provided insights into the flow routes and physiological effects of the internal hemolymph. However, the physical aspects of internal flow in the vein network, such as flow rate and pressure drop, have not been well studied.

Wing veins can be modeled as conduits or hydraulic resistors to form a circuit. Their hydraulic resistances are inversely proportional to the fourth power of the diameter and directly proportional to the length of the Poiseuille flow [9], and their arrangement determines the combined resistance of the entire vein network. From an engineering perspective, a reduction in network resistance improves the frictional pressure loss and power consumption for internal hemolymph flow. A previous study suggested that diameters of wing veins at network junctions in butterflies and moths follow Murray’s law, which is related to the minimization of total energy consumption owing to frictional loss and metabolism [10]. In our earlier research, the hemodynamic effects of venous networks in a fruit fly species, *Drosophila melanogaster*, was examined based on a fluid-mechanics simulation [11]. This shows that the presence of the posterior crossvein (PCV) improves the total frictional loss between the vein network inlet and outlet, functioning as a hydraulic resistor that is parallel to the resistors having the greatest resistance value. Examining these network adjustments in insects may aid in the development of networks for low-energy-consuming microfluidic devices. However, there is currently a lack of discussions on the hemodynamic effects of vein morphology and network topology in insect wings.

**Figure 1:**
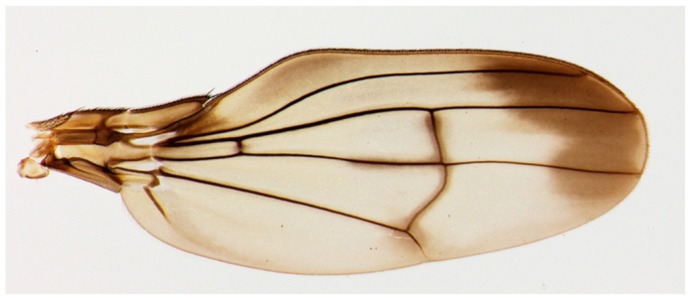
Actual photograph of a Hawaiian fruit fly species, *Drosophila cyrtoloma*, from Ref. [13].

**Figure 2:**
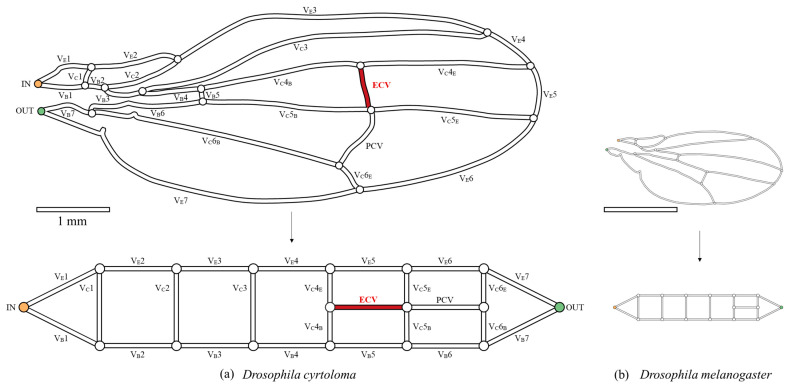
The network topology of the wing veins of fruit flies. (**a**) Hawaiian species with the extra crossvein (ECV), *Drosophila cyrtoloma*; (**b**) common fruit fly, *Drosophila melanogaster*. The upper figures are the schematic representations of the wing veins, with the vein lengths following the same 1 mm-scale bar. They were each scanned from digital photographs in previous publications [13,14]. The bottom panels show their topological models. The captioned numbers beginning with “V_E_” and “V_B_” beside the veins, respectively, indicate the edge veins and base veins. “V_C_” refers to the connecting veins, and the fourth, fifth, and sixth wing veins are divided into edge- and base-side segments, labeled “E” and “B”, respectively. The vein labeled “ECV” is the extra crossvein, highlighted in red, and another vein labeled “PCV” is the posterior crossvein. The orange and green circles, in the vein network diagram above, are the network inlet and outlet of hemolymph flow, respectively.

The vein networks of the forewings of a specific Hawaiian fruit fly group, the planitibia group, are of special interest because of their unique network topology, i.e., the forewing of *Drosophila cyrtoloma* in Figure 1 [12]. Most species in this group have an extra crossvein (ECV) [12,13], as highlighted in red in the schematic of the vein network in Figure 2a. This feature distinguishes them from common fruit flies of the *Drosophila* genus such as *D. melanogaster* [14], shown in Figure 2b, which share a common vein network topology, but which are lacking this vein. The ECV, contiguous with the common PCV, forms a cruciform structure within the network topology, as illustrated at the bottom of Figure 2a. It has been speculated that the ECV mechanically supports the wings [13], as the ECV-bearing flies exhibit large wing sizes [13,15] and elongated wing shapes [16]. However, the technical functions of this vein have not yet been investigated. 

*D. cyrtoloma*, a species found in Hawaii and belonging to the ECV-bearing group, exhibits a wing size that is 2.9 times larger in the length-dimension compared to *D. melanogaster*, a common species (Figure 2). Isometric scaling predicted a vein diameter that was also 2.9 times larger, but the actual mean vein diameter in this Hawaiian fly was 12% smaller, indicating hypoallometry [17]. This small diameter would decrease the moment of inertia of the wing, owing to the lower hemolymph mass, but increase hydraulic resistance and power consumption for circulation. In contrast, the thoracic length of *D. cyrtoloma* [18] was 5% greater than the isometric prediction based on that of *D. melanogaster* [19]. Because the wing hearts in *D. melanogaster* [20,21] are located in the thorax, an allometrically larger thorax in Hawaiian flies can accommodate large wing hearts. This is a morphological adaptation to counteract the higher pressure drop by generating greater suction pressure. 

Our hypothesis was that the unique presence of the ECV serves as a further adaptation to mitigate the increased hydraulic resistance caused by hypoallometric vein diameters. In this study, the hemodynamic effects of the presence and position of this vein were demonstrated. This vein was virtually removed or rearranged, and the hemolymph flow rate distribution and pressure loss were numerically simulated under steady state conditions. For the flow calculations, a circuit analogy was applied to the venous networks on the wings. This computational analysis is independent of the impractical physical vein manipulation in living insects, whereas this is not the case in observation-based approaches seen in conventional studies. Our analytical results showed that the presence of the ECV improved the frictional pressure loss of hemolymph flow in the insect wings.

## 2. Materials and Methods

### 2.1. Characterization of the Forewing of the Hawaiian Fly

The network morphology on the forewing of a Hawaiian fly species, *D. cyrtoloma*, was characterized to model hemolymph flow within this network. Seven vein segments lie along the edge of the wing (V_E_1–7), whereas the other seven lie along the base (V_B_1–7), as shown in Figure 2a. These sets of veins are called edge veins and base veins, respectively, in the present study. They correspond to the upper and bottom serial veins in our topological model, shown at the bottom of Figure 2a. These are connected by veins labeled V_C_1–6, which are called connecting veins. The two crossveins, the ECV (red vein in Figure 2a) and PCV, link two pairs of connecting veins and share their connections, creating a crossing point. 

The length, l_n_, and inner diameter, d_n_, of each vein were determined through image analysis of the forewing of D. cyrtoloma [13] by means of ImageJ software ver. 1.54g (courtesy of the National Institutes of Health, Bethesda, MD, USA). The spatial resolution of the original image, Figure 1, was 1.9 µm/pixel. The average of three measurements for each vein’s length was used. The mean value of the measurements of the outer diameter at five different points of each vein was employed. These parameters are listed in Table 1. The cross-section of the inner space of the veins was treated as circular, and its diameter d_n_ was defined as 20% of the measured outer diameter. These settings were based on the observation of the wing vein’s cross-section in D. melanogaster [22], which belongs to the Drosophila genus, as well as those in the Hawaiian fruit fly. The measured values of lengths and diameters of wing veins may include an average of 1.4% and 20% errors, respectively, which originate from the spatial resolution of the image analysis.

A value was defined for allometric scaling between the Hawaiian fly and the common fruit fly species, *D. melanogaster*. The wing area of the Hawaiian fly is 8.3 times larger than that of the common fruit fly, based on our measurements of digital photographs [13,14]. According to the isometric concept, the wing of the Hawaiian fly is *k* = 2.9 times as long. *k* was used as an allometric scaling value, indicating the extent to which the ECV-bearing wings compare with common wings.

### 2.2. Calculation of the Hemolymph Flow

Hemolymph flow in the network of wing veins was modeled as a Poiseuille flow in a two-dimensional network of cylindrical pipes. The average inner diameters of the veins are of the order of 10 µm, as shown in Table 1. The flow velocities are of the order of 100 µm/s in *Anopheles gambiae* [2], which belongs to Diptera order as the same as flies belong. The viscosity of the hemolymph, *μ*, is 1.3 × 10^−3^ Pa·s in *D. melanogaster* [23]. The density is at 1.02 × 10^3^ kg/m^3^, measured in a hawkmoth species, *Manduca sexta* [24], which is one the insects belonging to the Holometabola superorder, which is the same as that for flies. Consequently, the Reynolds number of this phenomenon is less than 10^−3^ indicating viscous flow [9]. The hemolymph of the adult fly was treated as a Newtonian fluid because hemolymph of adult moths belonging to Holometabola is recognized to be Newtonian [25]. 

According to Poiseuille’s law, the frictional pressure loss in a vein is as follows:

(1) $$∆p_{n}=\frac{{128\;\mu l}_{n}}{{\pi d}_{n}^{\;4}}q_{n},$$

at a volumetric flow rate of *q*_n_. The hydraulic resistance was defined as *r*_n_ = 128 *µl*_n_/*πd*_n_^4^, a constant value that depends on known parameters of the vein length and diameter, along with the fluid viscosity. Thus,

(2) $$∆p_{n}=r_{n}q_{n}.$$

The pressure losses and the flow rates in the wing veins were undetermined quantities in our calculation. This equation was simultaneously established for all wing veins in the network, treated as a planar hydraulic resistor system. 

The established equations form a matrix following the laws of mass and energy conservation, which correspond to Kirchhoff’s laws for electrical circuits. At each node, the inflow rate *q*_in_ and the outflow rate *q*_out_ are equal, i.e., *q*_in_ = *q*_out_, for conservation of the fluid mass. Over each closed loop of the veins, the sum of their pressure losses satisfies the energy conservation: ΣΔ*p*_n_ = Σ*r*_n_*q*_n_ = 0. In one closed loop that includes pressure sources, the sum of the pressure losses is equal to the sum of the pressure sources. Our network model contained one pressure source. Thus, in our model, the loop including the pressure source fulfills ΣΔ*p*_n_ = Δ*p*_total_ = Σ*r*_n_*q*_n_, in which Δ*p*_total_ is the total pressure loss. 

**Figure 3:**
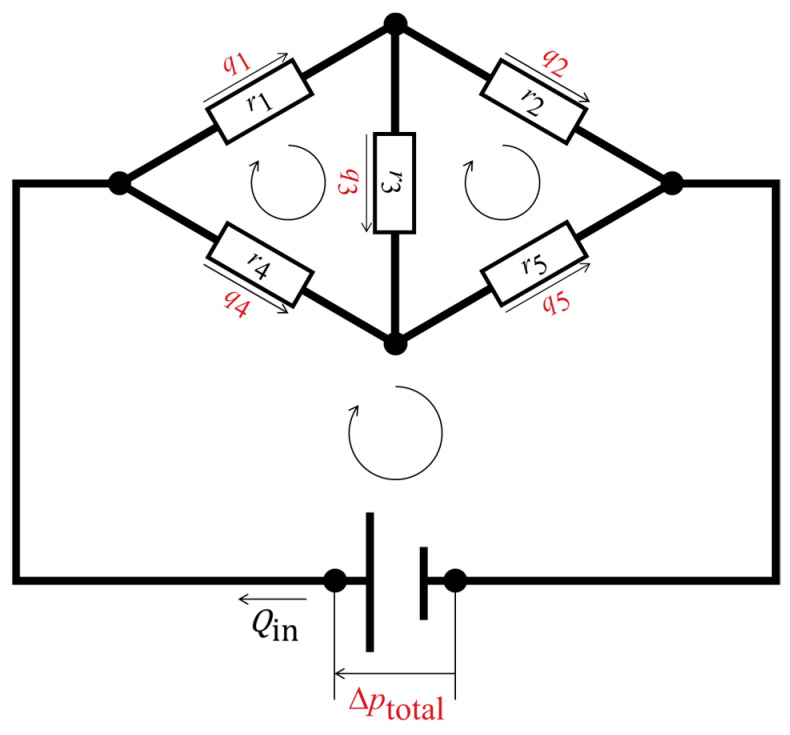
Schematic representation of a fluidic circuit with five hydraulic resistors and three closed loops. The black circles are nodal points at which the conservation of mass of the fluid is fulfilled. The hydraulic resistors are represented by the white rectangles. The assumed flow directions are shown as the straight arrows, with the volumetric flow rates; the closed loops are represented by the looped arrows, where the sums of the pressure losses are zero or equal to the source pressure. The undetermined physical quantities are highlighted in red, while the determined physical quantities are shown in black.

Figure 3 shows an example fluidic circuit containing five hydraulic resistors and three closed loops. The following matrix form was set up for this fluidic circuit based on a simultaneous equation system that satisfies the conservation laws:

(3) $$\left(\begin{array}{ccc} r_{1}+r_{3}+r_{4} & -r_{3} & 0\\ -r_{3} & r_{2}+r_{3}+r_{5} & 0\\ -r_{4} & -r_{5} & -1 \end{array}\right)\cdot \left(\begin{array}{c} q_{1}\\ q_{2}\\ ∆p_{\mathrm{total}} \end{array}\right)=\left(\begin{array}{c} r_{4}Q_{\mathrm{in}}\\ r_{5}Q_{\mathrm{in}}\\ -(r_{4}+r_{5})Q_{\mathrm{in}} \end{array}\right)$$

where *Q*_in_ represents the volumetric inflow rate of the network. It exhibits the *Ax* = *y* notation, where *A* is a matrix that includes the known resistance values and the notation 0 or −1, indicating the inclusion of pressure sources in the loops; the vector *x* contains the undetermined flow rates in the veins and the source pressure; and *y* includes the pressure loss determined by the network inflow rate and the hydraulic resistance values. The number of equations required for matrix formation must be equal to the number of unknown variables. The interdependent variables should be combined to minimize the number of equations (e.g., *q*_3_ = *q*_1_ − *q*_2_). Matrix equations for the network model of the wing veins were established according to the same concept. The linear matrices were solved using MATLAB (MathWorks, Natick, MA, USA) with double precision. The same calculation was performed in a previous report to derive hemolymph flow in a fruit fly species [11] and was applied to the microfluidics discipline [26,27,28].

The inflow and outflow rates, *Q*_out_, of the venous network were fixed at the boundary, *Q*_in_ = *Q*_out_ = 12 pl/s. This value is equivalent to *k*^3^ times the assumed hemolymph flow rate in *D. melanogaster* [11]. The inlet and outlet of hemolymph were set at the anterior and posterior veins at the base, as shown in Figure 2a. It was assumed that hemolymph filled all the wing veins of fruit flies, according to results from previous studies regarding the hemolymph paths of other Dipteran species [1,2]. 

In this flow calculation, minor losses were ignored, except for pressure loss due to internal friction, which is more than 10^5^ times greater than the other losses, based on the current Reynolds number [9]. The calculated hydraulic resistances and frictional losses within the wing veins may range from 0.48 to 2.5 times the true values because of the limited spatial resolution of the original image of the wing.

### 2.3. Network Modeling for the Repositioning of the ECV

**Figure 4:**
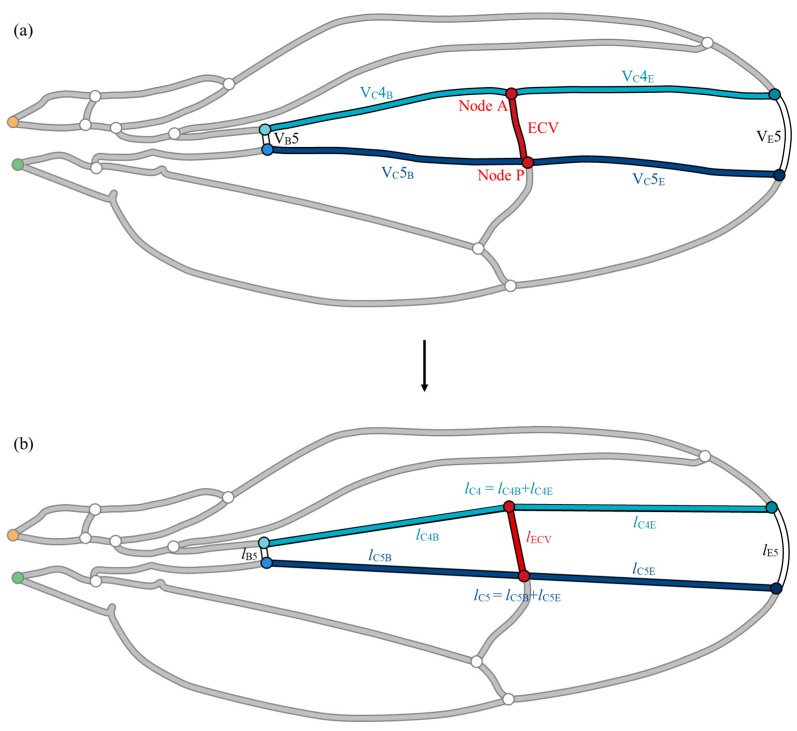
Simplification of the vein group surrounding the extra crossvein (ECV). (**a**) Actual set of veins; (**b**) simplified model of the vein set. The fifth edge vein, V_E_5, has been replaced with an arc, whereas the following veins were substituted by the straight line segments during the simplification: the fifth base vein, V_B_5; anteriorly and posteriorly contiguous connecting veins, V_C_4 and V_C_5. The circles labeled “Node A” and “Node P”, highlighted in red, are the connections of the ECV on the anterior and posterior sides, respectively. The orange and green circles are the network inlet and outlet of hemolymph flow, respectively.

To confirm the hemodynamic effects of the ECV position, parametric repositioning of the vein was required. Its anterior and posterior ends, nodes A and P, divide the fourth and fifth connecting veins-, respectively, as shown in Figure 4a. The positions of the connecting veins were virtually controlled to change the position of the ECV. To calculate the flow parameters corresponding to the positions, the lengths of the edge and base segments of the connecting veins and the ECV during node shifting must be determined. The vein network was geometrically simplified to avoid complexity in the derivation of varying vein lengths, as shown in Figure 4b. This simplified network model was used to demonstrate the positional effect of the ECV, while the effects of the presence of these veins were examined within the network model shown in Figure 4a, reflecting the measurements on the actual wing vein network.

For simplicity, the segments of veins that surround the ECV were replaced with an arc and straight line segments, as shown in Figure 4b. The edge vein segment was substituted with an arc connecting the ends of the actual segement. This arc was defined to minimize modeling errors in the length of the edge vein. The other surrounding vein segments were replaced with straight-line segments. The ECV was substituted with a straight line linking Nodes A and P. Each coordinate of the end of vein was determined through image analysis using ImageJ software. This simplified network model included errors in the vein lengths that were less than 2% compared with the measured vein lengths, except for V_B_5, which showed an error of 5.9%, owing to its short length. The hydraulic resistances included the same rate of error because of the fixed vein diameter during the simplification. The total pressure loss was overestimated by 0.1% in this simplified network owing to geometric errors, confirmed by comparing the total pressure losses in the network models with the actual geometry and simplified geometry. 

Nodes A and P were parametrically shifted in the simplified model to demonstrate their positional effects on the hemolymph flow. Their shifted positions were defined with the lengths of the base-side segments of the contiguous connecting veins *l*_C4B_ and *l*_C5B_ in Figure 4b. Both *l*_C4B_/*l*_C4_ and *l*_C5B_/*l*_C5_ were virtually varied from 0.001 to 0.999 in increments of 0.001, in which *l*_C4_ and *l*_C5_ are the entire lengths of the respective connecting veins. While the actual positions of the ECV correspond to *l*_C4B_/*l*_C4_ = 0.486 and *l*_C5B_/*l*_C5_ = 0.506, the simplified model contains +0.55% and −0.47% errors in these ratios, respectively. During the connection shifting, the diameter of the ECV is constant, while its length varies with the positions of its connections.

## 3. Results

### 3.1. Effect of the ECV’s Presence on Hemolymph Flow

In the absence of the ECV, hemolymph flowed from the inlet towards the outlet of the vein network through the series of edge and base veins. Most of the connecting veins exchange liquids between the series. A portion of the hemolymph bypassed the sixth edge and base veins and flowed into the PCV via segments of the fifth and sixth connecting veins. The total pressure loss between the inlet and outlet in this ECV-less network was 0.62 kPa. These flow pathways are the same as those in a common fruit fly without an ECV [11].

**Figure 5:**
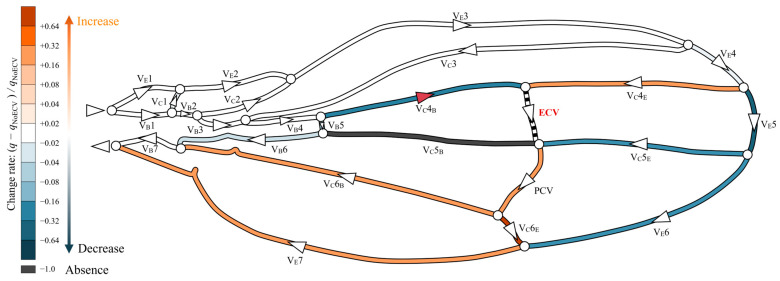
Change in flow rate distribution owing to the presence of the extra crossvein (ECV). The wing veins are colored based on their rates of change resulting from the comparison of their volumetric flow rates in the ECV’s presence and absence: (*q* − *q*_NoECV_)/*q*_NoECV_, where *q* is the flow rate in the presence of the ECV, and *q*_NoECV_ is that in its absence. Orange and blue, respectively, indicate an increase and a decrease in the flow rate. Black indicates no flow rate, or less than 0.1% of *Q*_in_. Faint and dark colors indicate small and large changes, respectively. The triangles on the veins indicate the direction of flow, and white circles are bifurcations of the veins. The red triangle on the V_C_4_B_ means that the flow direction has changed due to the presence of the ECV.

The presence of the ECV diverted some hemolymph from the fifth pair of edge and base veins through the fourth connecting vein segment, as shown in Figure 5. The ECV exhibited a volumetric flow rate of 0.14*Q*_in_ by draining the same total flow rate amount from the fifth pair of edge and base veins. The ECV and edge-side segment of the fifth connecting vein transferred the hemolymph to the PCV. In this crossvein, the flow rate increased by 30% compared to that without the ECV, causing the flow rates in the sixth vein pair to decrease. The base-side segment of the fifth connecting vein lost hemolymph flow. Overall, the presence of the ECV reduced the flow rate in the fifth and sixth pairs of the edge and base veins. The total pressure loss decreased by 3.1% compared to that without the ECV. 

### 3.2. Positional Effect of the ECV 

**Figure 6:**
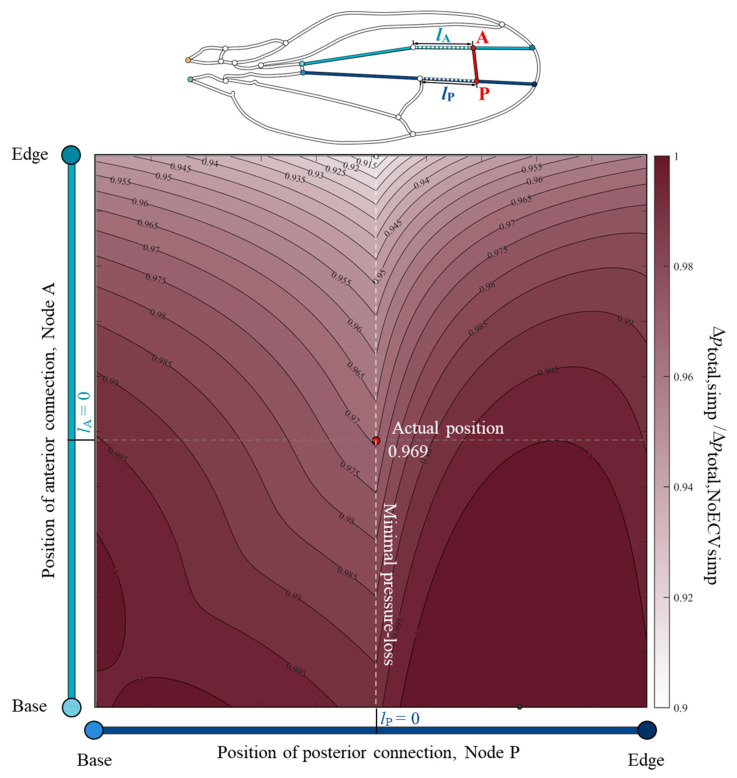
Normalized variation of the total pressure loss according to the positions of the connections of the extra crossvein (ECV) in the simplified vein network model. The vertical and horizontal axes show the positions of the anterior and posterior connections of the ECV, Nodes A and P. The points *l*_A_ = 0 and *l*_P_ = 0 on the respective axes mean that the nodes are located at the actual positions, where *l*_A_ and *l*_P_ are the respective distances between the nodes and their actual positions. The color intensity in the color map indicates the magnitude of the normalized pressure loss. The total pressure loss in the simplified vein network model, Δ*p*_total,simp_, is the normalized the pressure loss without the ECV, Δ*p*_total,NoECVsimp_. The red circle represents the actual position of the ECV. The black and white circles mark the positions at which the pressure loss is maximum and minimum, respectively. The orange and green circles, in the vein network diagram above, are the network inlet and outlet of hemolymph flow, respectively.

To clarify the relationship between the position of the ECV and its pressure-loss reducing effect, the total pressure drop was examined using the changed positions of the ECV. The color map, Figure 6, showed valley-like isovalue contours along the actual position of the posterior connection and a broad monotonic decrease along the vertical axis. This indicates that the total pressure loss decreases as the anterior connection approaches the edge. The posterior connection at the actual position showed minimal total pressure loss, while the anterior connection was fixed at an arbitrary position. For example, when the anterior connection was fixed at the actual position, the total pressure loss with the posterior connection at the actual position was minimal, which was 3.1% less than that without the ECV. The actual position of the posterior connection matched the anterior connection of the PCV. Therefore, the shared connection of the ECV and PCV minimized the total pressure loss. 

## 4. Discussion

### 4.1. How the Presence of the ECV Reduced the Total Pressure Loss

The presence of the ECV reduced the total pressure loss through the hemolymph flow in the vein network. The total pressure loss equals the sum of the local pressure losses in the edge or base vein series in the steady state, corresponding to Kirchhoff’s voltage law. These local pressure losses were determined by the individual hemolymph flow rates, as the resistance values of the vein members were constant. The ECV and PCV can be considered as hydraulic resistors parallel to the fifth and sixth pairs of edge and base veins, respectively, as shown in the topological model at the bottom of Figure 2a. These crossveins can reduce the flow rate in these vein pairs by diverting the hemolymph. The presence of the ECV directed some of the hemolymph to this vein instead of to its parallel vein pair and increased the PCV’s flow rate. Increasing the flow rate of the PCV decreased the flow rate in the sixth edge and base veins. This enhanced the reduction in the total pressure loss, as this pair of veins exhibited the greatest resistance value compared with all other vein pairs, as observed in common fruit fly species [11]. The ECV, together with the PCV, formed a resistor compound that divided the hemolymph flow and reduced the total pressure loss. 

**Figure 7:**
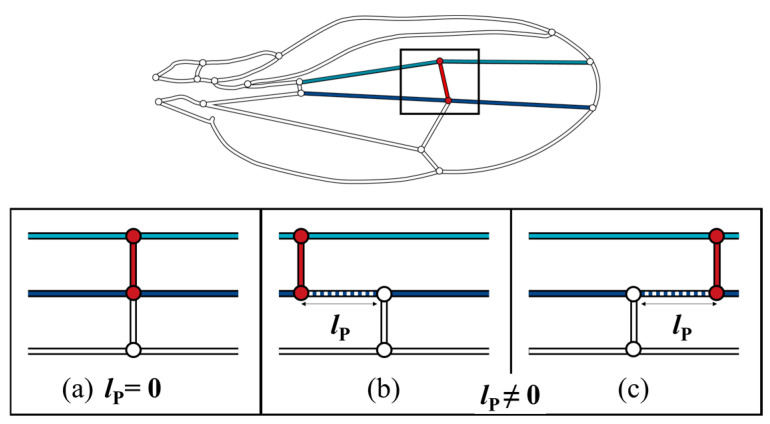
Three scenarios of different *l*_P_, pathway length, between the extra crossvein (ECV) and the posterior crossvein (PCV). Light blue and navy line segments are the connecting veins that are the anteriorly and posteriorly contiguous to the ECV, respectively. (**a**) the ECV and PCV are contiguous as *l*_P_ = 0; (**b**,**c**) these two crossveins are not contiguous, *l*_P_ ≠ 0, where the ECV is more proximally and distally positioned, respectively.

The color map in Figure 6 shows that the pressure loss was minimal when the ECV was directly connected to the PCV. When they are contiguous, the bypassing hemolymph in the ECV flows directly into the other crossvein, as shown in Figure 7a, in which the pathway from the ECV to PCV has no length, *l*_P_ = 0, and no pressure loss occurs between them. However, when their connections were far apart (*l*_P_ ≠ 0), as shown as Figure 7b,c, another vein segment transfers hemolymph between them, causing extra pressure loss that is proportional to *l*_P_. The ECV’s posterior connection slightly shifts from the position with *l*_P_ = 0, and the extra pressure loss occurs, resulting in the discontinuous pressure loss gradient. The actual position of the posterior connection of the ECV ensures contiguity of these crossveins and minimizes this additional pressure loss to zero. This has a pressure loss-reducing effect on the ECV-bearing network.

### 4.2. Demonstration of the Hemodynamic Effects of the Presence of the ECV in Idealized Circuits

**Figure 8:**
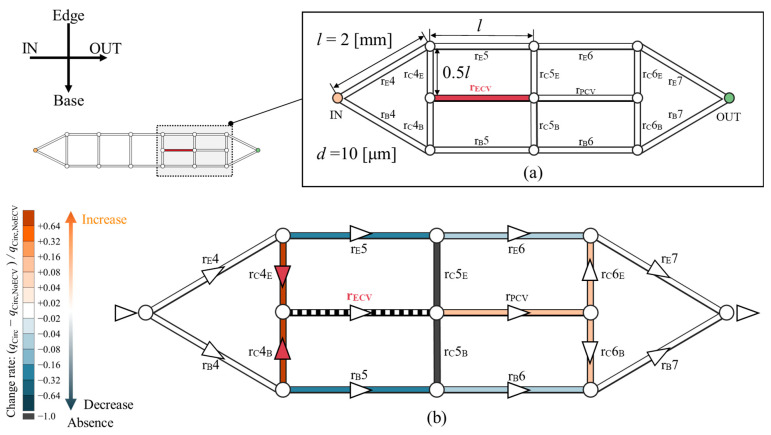
Changes in hemolymph flow owing to the presence of the extra crossvein resistor (ECV resistor) in an idealized circuit model imitating the vein network topology of the Hawaiian fly’s wing. (**a**) Idealized circuit model; (**b**) change in the flow rate distribution across the circuit because of the presence of the ECV resistor. Resistors are colored according to their change rates, (*q_Circ_* − *q*_Circ,NoECV_)/*q_Circ,_*_NoECV_, where *q_Circ_* is the flow rate in the presence and *q_Circ,_*_NoECV_ is that in the absence of the ECV resistor. Orange and blue, respectively, indicate an increase and a decrease in the flow rate. Black indicates no flow rate, or less than 0.1% of *Q*_in_. Faint and dark colors indicate small and large changes, respectively. The triangles on the veins represent the flow directions, and white circles are bifurcations of the veins. The red triangles mean that the hemolymph is flowing due to the presence of the ECV.

The hemodynamic effect of the ECV was computationally tested within an idealized circuit model, as shown in Figure 8. The shape of this circuit model is based on the network topology of the wing veins of the Hawaiian fly. It represents the fourth to seventh sections of the vein network containing the ECV and PCV (Figure 8a). The circuit model consists of microchannels. They are treated as hydraulic resistors with corresponding resistance values, and they were named after their corresponding vein segments. r_E_, r_B_, and r_C_ are the edge, base, and connecting resistors, respectively; and r_ECV_ and r_PCV_ are the ECV and PCV resistors, respectively. All channels were 2 mm long, with the exception of the connecting resistors, which were half as long. Their diameter is uniformly 10 µm. These geometric settings originate from the mean values of the measured vein lengths and diameters with one significant digit. The flow rate of *Q*_in_ was provided to the inlet of the circuit model. The changes in hemolymph flow were examined after the removal and rearrangement of the ECV resistor.

In the absence of the ECV resistor, the PCV resistor exhibited 0.25 *Q*_in_ of the bypassed flow rate. In its presence, the ECV resistor diverted 0.29 *Q*_in_ of flow rate from the fifth pair of the resistors on the edge- and base-sides. The fifth connecting resistor lost hemolymph flow, as hemolymph flow was absent in the base-side segment of the fifth connecting vein in the actual vein network (Figure 5). The PCV resistor’s flow rate increased to 0.29 *Q_in_* owing to hemolymph transfer from the ECV resistor. The total pressure loss was 8.6% lower than that without the ECV resistor. These results characterize the hemodynamic effects of the presence of the ECV. 

**Figure 9:**
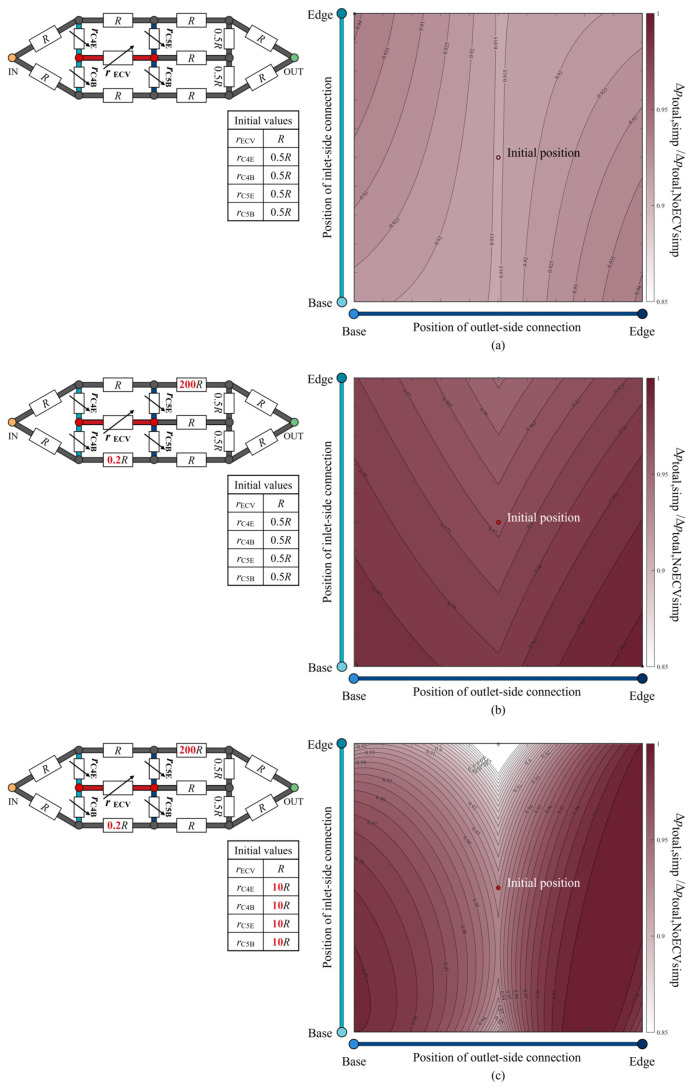
The normalized change in total pressure loss according to the position of the extra crossvein (ECV) in the idealized circuit models. The three scenarios considered are: (**a**) no change in the resistance values; (**b**) asymmetric distribution of the resistance values; (**c**) increased resistance of the fourth and fifth connecting resistors, in addition to the asymmetric resistance distribution. Each schematic of the circuit model at the top left of each color map shows the resistance values of the resistors. These values are normalized by the initial resistance value of the ECV resistor, *R*. The symbols of the variable resistors for the fourth and fifth connecting resistors and the ECV resistor indicate that their resistance values vary, depending on the positions of the ECV resistor’s connections. The initial resistance values of these variable resistors are shown in the bottom of the respective circuit diagrams. The vertical and horizontal axes show the positions of the inlet-side and outlet-side of the connections. The color intensity in the color map indicates the level of the normalized total pressure loss, Δ*p*_total,circ/_Δ*p*_total,NoECVcirc_, where Δ*p*_total,circ_ is the total pressure loss corresponding to the ECV-resistor’s position, and Δ*p*_total,NoECVcirc_ is that without the ECV resistor. The red circle represents the initial position of the ECV resistor. The black and white circles mark the positions at which the pressure loss is maximum and minimum, respectively. In the color map (**a**), the initial position and the minimum position coincide.

The total pressure loss when varying the positions of the ECV resistor was examined and is shown as a color map in Figure 9a. The hydraulic resistances of the fourth and fifth connecting resistors and the ECV resistor were variables, as their lengths varied with the positions of the connections of the ECV resistor. The variable resistances can be written as functions of their lengths with fixed diameters, according to Equations (1) and (2). The resistance values of the connecting resistors were set to *r*_C_ = 0.5*R* at the initial position of the ECV resistor, where that of the ECV resistor at its initial position was *r*_ECV_ = *R* because the initial lengths of the connecting resistors were half the length of the ECV resistor, as shown in Figure 8a. Their diameters were constant during the virtual rearrangement.

The pressure loss was minimized by placing the ECV resistor in the initial position inside the simplified circuit, where all adjacent connecting resistors exhibited equal lengths or resistance values, as shown in Figure 9a. For any fixed position of the inlet-side connection, the pressure loss was minimized when the outlet-side connection was contiguous with the PCV resistor, as seen in the actual vein network (Figure 6). This result shows that the shortest connection between the ECV and the PCV provides minimal total pressure loss. 

The isovalue contours of the pressure-loss color map exhibited symmetrical variations with respect to the initial position, as shown in Figure 9a, but no valley-like variation, unlike that of the actual vein network shown in Figure 6. We investigated the characteristics of the resistance distribution across the wing vein network, which determines the total pressure loss variation according to the position of the ECV. Although our idealized circuit model had symmetric resistance distribution, the actual wing vein network was characterized by an asymmetric distribution. The diameters of the fifth base and the sixth edge resistor in the idealized circuit were modified to obtain 0.2*R* and 200*R* of resistance values, respectively. The resistance settings were determined based on the highest and lowest resistance values found in the fourth to seventh portions of the actual vein network: the resistance values of the fifth base vein and the sixth edge vein, respectively. The way in which this asymmetrical resistance distribution affects the isovalue contours was tested in an idealized circuit, as shown in Figure 9b. It shows valley-like isovalue contours, with minimum values along the centerline. The valley-like contours depend on the asymmetry of the resistance distribution, with high resistances in the edge vein series and low resistances in the base vein series. 

Which vein resistance influences the steepness of the valley contours? In the actual vein network, the connecting veins contiguous to the crossveins exhibit a resistance that is, on average, 10 times greater than the ECV with one significant digit. The diameters of the fourth and fifth connecting resistors were changed to set their resistance values to 10*R,* with the initial position of the ECV resistor in the asymmetrical circuit. Figure 9c shows the pressure loss variation in the redesigned circuit with the increased resistance of the connecting resistors. The isovalue contours show a steeper valley-like variation than that shown in Figure 9b. Higher resistances may increase the pressure loss in hemolymph transfer between the ECV and PCV resistors when their connections are not contiguous. Therefore, the characteristics of pressure loss variation due to the ECV’s position may depend on the contiguity of the ECV and PCV, the asymmetric distribution of the hydraulic resistances across the network, and the large resistance in the connecting vein segments relative to the ECV.

Our results show that the presence of the ECV reduces the total pressure loss between the inlet and outlet of the wing vein network. This may mitigate the increase in resistance due to the allometrically small vein diameters of ECV-bearing wings. The hyperallometric thorax length of the Hawaiian fly is another morphological adaptation that compensates for the increased network resistance by housing an allometrically larger wing heart. A small vein diameter may reduce the internal vein volume and internal hemolymph mass within the wing. This theoretically leads to a reduction in the moment of inertia and power consumption during the flapping motion to accommodate the elongated and enlarged wing shapes of Hawaiian flies with the ECV. The presence of this vein could enable hemolymph circulation in the vein network with allometrically small diameters for morphological adjustment as a flying device, aided by the increased pressurized output of the wing heart. 

The position of the ECV is related to the minimization of pressure loss or power consumption owing to fluidic frictional loss. The results of our research can be used as a catalyst for the development of effective systems for transporting fluids. Certain microfluidic networks, such as fluidic radiator systems in microelectronic devices and microfluidics in lab-on-chip systems, require power in order to counteract the effects of frictional loss [26,28]. Implementing wing vein network topologies can enhance hydraulic resistance and reduce power consumption. 

In the present study, hemolymph circulation in the stationary state was demonstrated, but during flight, the wing flapping motion and aerodynamic load on the wing may influence internal hemolymph flow. A previous study reported that the flapping motion accelerates hemolymph flow within the wing of the dragon fly, taking advantage of the wing tip moving with an 8-shaped trajectory and the wing plane rotating around the torsional wing axis [29]. Flies are also known to exhibit the 8-shaped wing-tip motion and torsional rotation during the wing movement [30]. Pressure requirements for the wing heart in the Hawaiian fly may additionally decrease during their flight due to their wing kinematics. Flapping wings encounter an aerodynamic load unevenly distributed on the wing surface [31], and it can locally bend the wing veins. The inertial losses of hemolymph pressure, i.e., bend losses, are negligible due to the low Reynolds number, being 10^−5^ times that of the frictional losses. Therefore, local bending of the wing under a heterogenous aerodynamic load would not influence the hemolymph flow distribution and the pressure-loss reducing effect incurred by the ECV’s presence. However, if the cross-sectional area of the veins periodically deforms during the flapping motion, the hydraulic diameter of the vein changes. This possibly influences the hemolymph flow rate distribution and pressure loss reducing effect.

The presence of the ECV may eventually increase wing weight and the wing’s moment of inertia, although it decreases the total pressure loss. The wing weight of the Hawaiian fly was estimated from the common fruit fly’s wing weight, 2.7 μg [32]. Considering this isometric relationship, the Hawaiian fly’s wing weight was estimated to be 65 μg without the ECV. The ECV’s mass is the sum of the internal hemolymph mass and the vein wall mass. The hemolymph mass equals the inner volume times the hemolymph density, 2.8 × 10^−2^ μg. The mass of the vein wall is equivalent to the residual volume of the ECV times the wing cuticle density, 1150 kg/m^3^ [33]; subsequently, the vein wall mass is estimated to be 0.77 μg. It was worth increasing the wing mass by 1.2%, although the pressure loss reduction rate was 3.1%. Additionally, the ECV’s presence may allow for the constriction of the vein diameters, leading to a decrease in hemolymph mass in the other veins, according to our results. Therefore, the ECV’s presence conceivably counterbalances the increase in pressure loss due to constricted vein diameter; even a slight increase in the wing mass is taken account.

Mathematical modeling of the pressure-loss reducing effect of the network topology with the ECV promotes these practical applications. The results shown in Figure 9 show that the ECV’s effects vary with the distribution of the hydraulic resistances across the networks. To predict the improvement of energy efficiency due to the ECV-imitating channels, the effect of the ECV’s presence needs to be theorized, corresponding to the resistance distribution across the network in the future.

## 5. Conclusions

The presence of the ECV in the wing of the Hawaiian fruit fly reduced the total pressure loss during hemolymph transportation within the vein network by 3.1%. This vein functions in conjunction with the posterior crossvein and serves as a parallel hydraulic resistor. The hemolymph flow rate decreased in the edge and base side vein segments because the hemolymph was able to bypass them and flow into the ECV and PCV. These crossveins formed a direct connection that minimized the total pressure loss; the same effect was confirmed in an idealized circuit model. These characteristics may mitigate the influence of allometrically small vein diameters on hydraulic resistance. This research provides practical insights into possible future transport network designs to minimize energy losses.

## Figures and Tables

**Table 1:** Geometries of the wing veins of *D. cyrtoloma*. The measured length, *l*_n_, and the outer diameter and calculated inner diameter, *d*_n_, of each wing vein (measured with ImageJ) are listed.

Vein	Length: *l*_n_ [mm]		Outer Diameter [mm]		Inner Diameter: *d*_n_ [mm]
V_E_1	8.9 × 10^−1^	±0.028	7.6 × 10^−2^	±0.028	1.5 × 10^−2^
V_E_2	1.2 × 10^0^	±0.0092	6.0 × 10^−2^	±0.038	1.2 × 10^−2^
V_E_3	4.5 × 10^0^	±0.0024	5.5 × 10^−2^	±0.012	1.1 × 10^−2^
V_E_4	7.7 × 10^−1^	±0.0050	4.3 × 10^−2^	±0.012	8.6 × 10^−3^
V_E_5	7.4 × 10^−1^	±0.0017	2.6 × 10^−2^	±0.0027	5.2 × 10^−3^
V_E_6	2.7 × 10^0^	±0.0088	1.5 × 10^−2^	±0.0015	2.9 × 10^−3^
V_E_7	4.9 × 10^0^	±0.038	1.9 × 10^−2^	±0.0074	3.8 × 10^−3^
V_B_1	5.8 × 10^−1^	±0.0022	1.6 × 10^−1^	±0.013	3.2 × 10^−2^
V_B_2	2.5 × 10^−1^	±0.0019	1.4 × 10^−1^	±0.040	2.8 × 10^−2^
V_B_3	5.5 × 10^−1^	±0.015	3.5 × 10^−2^	±0.0057	7.0 × 10^−3^
V_B_4	8.3 × 10^−1^	±0.0057	4.2 × 10^−2^	±0.0029	8.3 × 10^−3^
V_B_5	1.6 × 10^−1^	±0.0026	4.0 × 10^−2^	±0.0046	7.9 × 10^−3^
V_B_6	1.6 × 10^0^	±0.031	3.5 × 10^−2^	±0.0020	7.0 × 10^−3^
V_B_7	7.2 × 10^−1^	±0.042	5.2 × 10^−2^	±0.016	1.0 × 10^−2^
V_C_1	2.6 × 10^−1^	±0.024	1.1 × 10^−1^	±0.042	2.3 × 10^−2^
V_C_2	1.2 × 10^0^	±0.0017	7.9 × 10^−2^	±0.029	1.6 × 10^−2^
V_C_3	5.0 × 10^0^	±0.015	3.7 × 10^−2^	±0.0073	7.4 × 10^−3^
V_C_4_E_	2.4 × 10^0^	±0.016	3.0 × 10^−2^	±0.0058	6.1 × 10^−3^
V_C_4_B_	2.3 × 10^0^	±0.0045	4.8 × 10^−2^	±0.0067	9.5 × 10^−3^
V_C_5_E_	2.3 × 10^0^	±0.0047	2.3 × 10^−2^	±0.0019	4.6 × 10^−3^
V_C_5_B_	2.3 × 10^0^	±0.0047	3.0 × 10^−2^	±0.0050	6.0 × 10^−3^
V_C_6_E_	4.4 × 10^−1^	±0.0096	2.1 × 10^−2^	±0.0095	4.2 × 10^−3^
V_C_6_B_	3.5 × 10^0^	±0.0046	3.0 × 10^−2^	±0.0070	6.0 × 10^−3^
ECV	6.2 × 10^−1^	±0.0038	3.7 × 10^−2^	±0.0036	7.4 × 10^−3^
PCV	9.3 × 10^−1^	±0.0061	3.5 × 10^−2^	±0.0029	7.0 × 10^−3^

## Data Availability

All relevant data are contained within the paper.

## References

[1] Arnold J.W. (1964). Blood Circulation in Insect Wings. Mem. Entomol. Soc. Can..

[2] Chintapalli R.T.V., Hillyer J.F. (2016). Hemolymph Circulation in Insect Flight Appendages: Physiology of the Wing Heart and Circulatory Flow in the Wings of the Mosquito *Anopheles gambiae*. J. Exp. Biol..

[3] Salcedo M.K., Jun B.H., Socha J.J., Pierce N.E., Vlachos P.P., Combes S.A. (2023). Complex Hemolymph Circulation Patterns in Grasshopper Wings. Commun. Biol..

[4] Pass G., Tögel M., Krenn H., Paululat A. (2015). The Circulatory Organs of Insect Wings: Prime Examples for the Origin of Evolutionary Novelties. Zool. Anz. J. Comp. Zool..

[5] Hillyer J.F., Pass G. (2020). The Insect Circulatory System: Structure, Function, and Evolution. Annu. Rev. Entomol..

[6] Salcedo M.K., Socha J.J. (2020). Circulation in Insect Wings. Integr. Comp. Biol..

[7] Mengesha T.E., Vallance R.R., Mittal R. (2011). Stiffness of Desiccating Insect Wings. Bioinspir. Biomim..

[8] Lietz C., Schaber C.F., Gorb S.N., Rajabi H. (2021). The Damping and Structural Properties of Dragonfly and Damselfly Wings during Dynamic Movement. Commun. Biol..

[9] Munson B.R. (2002). Fundamentals of Fluid Mechanics.

[10] Schachat S.R., Boyce C.K., Payne J.L., Lentink D. (2021). Lepidoptera Demonstrate the Relevance of Murray’s Law to Circulatory Systems with Tidal Flow. BMC Biol..

[11] Sugiyama K., Kubota Y., Mochizuki O. (2024). Circuit Analogy Unveiled the Haemodynamic Effects of the Posterior Cross Vein in the Wing Vein Networks. PLoS ONE.

[12] Edwards K.A., Doescher L.T., Kaneshiro K.Y., Yamamoto D. (2007). A Database of Wing Diversity in the Hawaiian *Drosophila*. PLoS ONE.

[13] Carson H.L., Clayton F.E., Stalker H.D. (1967). Karyotypic Stability and Speciation in Hawaiian *Drosophila*. Proc. Natl. Acad. Sci. USA.

[14] Torquato L.S., Mattos D., Matta B.P., Bitner-Mathé B.C. (2014). Cellular Basis of Morphological Variation and Temperature-Related Plasticity in *Drosophila melanogaster* Strains with Divergent Wing Shapes. Genetica.

[15] Church S.H., Extavour C.G. (2022). Phylotranscriptomics Reveals Discordance in the Phylogeny of Hawaiian *Drosophila* and *Scaptomyza* (Diptera: Drosophilidae). Mol. Biol. Evol..

[16] Spieth H.T. (1978). Courthip Patterns and Evolution of *Drosophila adiastola* and *planitibia* Species Subgroup. Evolution.

[17] Shingleton A.W., Mirth C.K., Bates P.W. (2008). Developmental Model of Static Allometry in Holometabolous Insects. Proc. R. Soc. B Biol. Sci..

[18] Craddock E.M., Kambysellis M.P., Franchi L., Francisco P., Grey M., Hutchinson A., Nanhoo S., Antar S. (2018). Ultrastructural Variation and Adaptive Evolution of the Ovipositor in the Endemic Hawaiian Drosophilidae. J. Morphol..

[19] Santos M., Fowler K., Partridge L. (1994). Gene–Environment Interaction for Body Size and Larval Density in *Drosophila melanogaster*: An Investigation of Effects on Development Time, Thorax Length and Adult Sex Ratio. Heredity.

[20] Tögel M., Pass G., Paululat A. (2013). In Vivo Imaging of *Drosophila* Wing Heart Development during Pupal Stages. Int. J. Dev. Biol..

[21] Tögel M., Meyer H., Lehmacher C., Heinisch J.J., Pass G., Paululat A. (2013). The bHLH Transcription Factor Hand Is Required for Proper Wing Heart Formation in *Drosophila*. Dev. Biol..

[22] Steinbrecht R.A. (1993). Freeze-Substitution for Morphological and Immunocytochemical Studies in Insects. Microsc. Res. Tech..

[23] Zabihihesari A., Parand S., Rezai P. (2023). PDMS-Based Microfluidic Capillary Pressure-Driven Viscometry and Application to *Drosophila melanogaster*’s Hemolymph. Microfluid. Nanofluid..

[24] Kenny M.C., Giarra M.N., Granata E., Socha J.J. (2018). How Temperature Influences the Viscosity of Hornworm Hemolymph. J. Exp. Biol..

[25] Brasovs A., Palaoro A.V., Aprelev P., Beard C.E., Adler P.H., Kornev K.G. (2023). Haemolymph Viscosity in Hawkmoths and Its Implications for Hovering Flight. Proc. R. Soc. B Biol. Sci..

[26] Oh K.W., Lee K., Ahn B., Furlani E.P. (2012). Design of Pressure-Driven Microfluidic Networks Using Electric Circuit Analogy. Lab Chip.

[27] Gompper G., Fedosov D.A. (2016). Modeling Microcirculatory Blood Flow: Current State and Future Perspectives. WIREs Syst. Biol. Med..

[28] Rousset N., Lohasz C., Boos J.A., Misun P.M., Cardes F., Hierlemann A. (2022). Circuit-Based Design of Microfluidic Drop Networks. Micromachines.

[29] Wang Y., Yin Y., Zheng G., Yao H. (2020). Driving Mechanism of Dragonfly’s Wing Flapping Pattern for Liquid Circulation inside Wing. Anim. Biol..

[30] Muijres F.T., Elzinga M.J., Melis J.M., Dickinson M.H. (2014). Flies Evade Looming Targets by Executing Rapid Visually Directed Banked Turns. Science.

[31] Chen L., Wu J. (2024). Coexistence of dual wing–wake interaction mechanisms during the rapid rotation of flapping wings. J. Fluid Mech..

[32] Bergou A.J., Ristroph L., Guckenheimer J., Cohen I., Wang Z.J. (2010). Fruit Flies Modulate Passive Wing Pitching to Generate In-Flight Turns. Phys. Rev. Lett..

[33] Vincent J.F.V., Wegst U.G.K. (2004). Design and Mechanical Properties of Insect Cuticle. Arthropod Struct. Dev..

